# Monofilament anti-rotational suture combined with TPLO to prevent pivot shift: surgical technique and novel TPLO plate design

**DOI:** 10.3389/fvets.2025.1456869

**Published:** 2025-09-18

**Authors:** Dirsko J. F. von Pfeil, Parker N. House

**Affiliations:** ^1^Small Animal Surgery Locum PLLC, Dallas, TX, United States; ^2^Iowa State University College of Veterinary Medicine, Ames, IA, United States

**Keywords:** dog, cranial cruciate ligament rupture, tibial plateau leveling osteotomy, pivot shift, anti-rotational suture

## Abstract

**Objectives:**

To identify dogs at risk of developing pivot-shift (PS) following tibial-plateau-leveling-osteotomy (TPLO) using a rotational-instability-test (RI-test), describe a combination of a monofilament anti-rotational suture (ARS) with TPLO and assess this technique for feasibility, compare post-operative PS-incidence in dogs receiving a standard TPLO (TPLO-only) or a TPLO with ARS (TPLO+ARS), and design a novel TPLO-plate facilitating ARS-anchoring (TPLO/ARS-plate).

**Study design:**

In this clinical pilot trial on 85 client-owned dogs and instrumentation design study, the RI-test and ARS-placement-technique were described and performed. Reporting included: anesthesia and surgery times, bone-healing, post-TPLO-PS-incidence, follow-up and complications. Significance was set as *p* ≤ 0.05. Comprehensive engineering of a novel TPLO plate was performed.

**Results:**

Between TPLO-only (*n* = 57) and TPLO+ARS (*n* = 28) groups, significant differences were found for surgery time (*p* = 0.01), anesthesia time (*p*<0.001) and bone healing scores (*p* = 0.03), all being longer/higher for TPLO+ARS. PS-incidence was 2/57 (TPLO-only) and 0/28 (TPLO+ARS) within the first 8 weeks post-surgery (*p* = 1.00). Medium follow-up was 642 days. Major complications during that time occurred in 2/57 (TPLO-only; infection and implant removal) and 1/28 (TPLO+ARS; infection, PS-development and implant removal) dogs (*p* = 1.00). A novel TPLO/ARS-plate was designed.

**Conclusion:**

Post-TPLO-PS might be reduced following ARS placement. Additional studies are indicated to validate and refine the RI-test and assess the novel TPLO/ARS-plate in the clinical setting.

## 1 Introduction

Canine cranial cruciate ligament rupture (CCLR), resulting in cranial displacement and rotational tibial instability, is frequently addressed via tibial-plateau-leveling-osteotomy (TPLO), typically providing excellent clinical outcome (1–5). Remaining femoro-tibial instability is reported to persist in up to 33% of cases (6–8), including internal tibial rotation, (8), which has been suggested as an important etiology for the pivot-shift-phenomenon (PS) (2, 3, 8–12). Other discussed reasons for PS include a lack of periarticular fibrosis in hyperlax stifles, meniscal injury, meniscectomy, meniscal release or angular limb deformities (3, 6, 9, 13–16). The characteristic pivot-shift gait abnormality, described as internal stifle rotation/external hock rotation during the stance phase, is associated with rotational stifle instability (10). Persistent instability may result in meniscal injury, cartilage erosion and the progression of osteoarthritis, suggesting that treatment to prevent or manage this instability should be considered (10, 11, 16, 17).

Placement of an anti-rotational suture (ARS) following TPLO has been reported as both a method of treatment for and prevention of PS by controlling internal tibial rotational instability (10, 15, 16). It functions similar to a standard lateral-fabellotibial-suture (18). One reported ARS-material is a multifilament, braided ultra-high molecular weight polyethylene synthetic suture (UHMWPE) running between a femoral bone anchor and a specific TPLO-plate (10, 16). This material has been associated with bone-tunnel widening, local tissue irritation, stability loss including breakage at the anchor hole, chronic irritation and infection; the latter perceived to be as high as 84% when utilized to treat CCLR (5, 19–22). An alternative ARS-material, monofilament suture, threaded from the fabella through a tibial bone tunnel, has a perceived reduced infection risk (5), but was associated with 17.4% major complications when used as ARS, including tibial tubercle fracture (15). To minimize this risk, the suture could be led over the cranial aspect of the tibial tuberosity and anchored around the TPLO plate or TPLO-locking screw, instead of being threaded through a tibial bone tunnel. However, such ARS-anchoring might present technical challenges and potentially carry the inherent risk of suture breakage. To facilitate ARS-anchorage and potentially reduce such complications, a novel TPLO-plate for future use should be designed.

The authors have diagnosed and confirmed severe internal rotational instability in dogs exhibiting pivot shift as reported (10). They have used a rotational instability test (RI-test) over the past decade as a standard component of their clinical examinations. Although not validated, this test appears very useful for assessing stifle internal instability and as it can indicate the need for ARS-placement, was deemed useful for the purpose of this study. In addition, the authors have observed that dogs presenting with post-TPLO-PS typically exhibited high tibial rotational instability when applying the RI-test (unreported data).

A comparison between dogs receiving a standard TPLO (TPLO-only) with dogs undergoing TPLO with addition of an ARS (TPLO+ARS) may demonstrate the value of an ARS to reduce PS. The goals of this study were to (1) prospectively compare the outcome of dogs grouped to TPLO-only or TPLO+ARS based on RI-test results, (2) describe the above introduced technique of monofilament ARS-placement in conjunction with TPLO, (3) report the outcomes and complications, including the incidence of PS, in both groups, (4) develop a novel TPLO-plate design, facilitating ARS-anchorage. Our hypotheses were that (1) the RI-test would help to provide a guideline to identify dogs at risk to develop PS, (2) ARS-placement would be feasible and reduce incidence of PS, (3) no significant differences in complications would be found between groups, (4) a novel TPLO-plate could be designed.

## 2 Materials and methods

### 2.1 Inclusion and exclusion criteria; establishment of sample size

Dogs undergoing TPLO between January 01, 2020 to January 01, 2022 had to meet the following inclusion criteria: unilateral stifle injury due to complete CCLR, TPLO-surgery without (TPLO-only) or with ARS (TPLO+ARS) using a standard or broad 3.5 mm TPLO-plate, an 8-week direct follow-up examination with radiographs, a minimum of 1-year follow-up via telephone call. Recorded data included signalment, history, examination findings, surgical details, radiographs, evidence of PS at the time of first radiographic recheck, complications until up to a minimum of 1 year. No evidence of palpable or radiographic stifle joint effusion and no instability with either instability test was mandatory in the contralateral stifle to serve as control.

Exclusions applied to dogs with partial or bilateral CCLR, limb deformities, any additional procedures performed during TPLO-surgery, bilateral stifle arthroscopies or the use of implants other than 3.5 standard or broad TPLO-plates or monofilament suture.

Considering the low PS-incidence (0.3–3.1%) (2, 3, 12), power analysis indicated that, to achieve 80% power, each group would necessitate 257 dogs (23). Therefore, it was decided to perform a pilot study first, targeting a minimum of 25 dogs per group. Since similar TPLO-ARS combinations had been reported (10, 15, 16), and the used technique represented a modification of those, approval from the institutional animal care and use committee was deemed unnecessary.

### 2.2 Diagnostics prior to TPLO-surgery and screening for “at-risk-dogs”

All dogs underwent complete blood count and serum chemistry testing, along with bilateral orthogonal stifle radiographs. A pre-operative diagnosis of complete CCLR was made through the cranial draw test as well as the tibial-compression-test, and then confirmed during surgery through mini-arthrotomy or arthroscopy.

**Figure 1:**
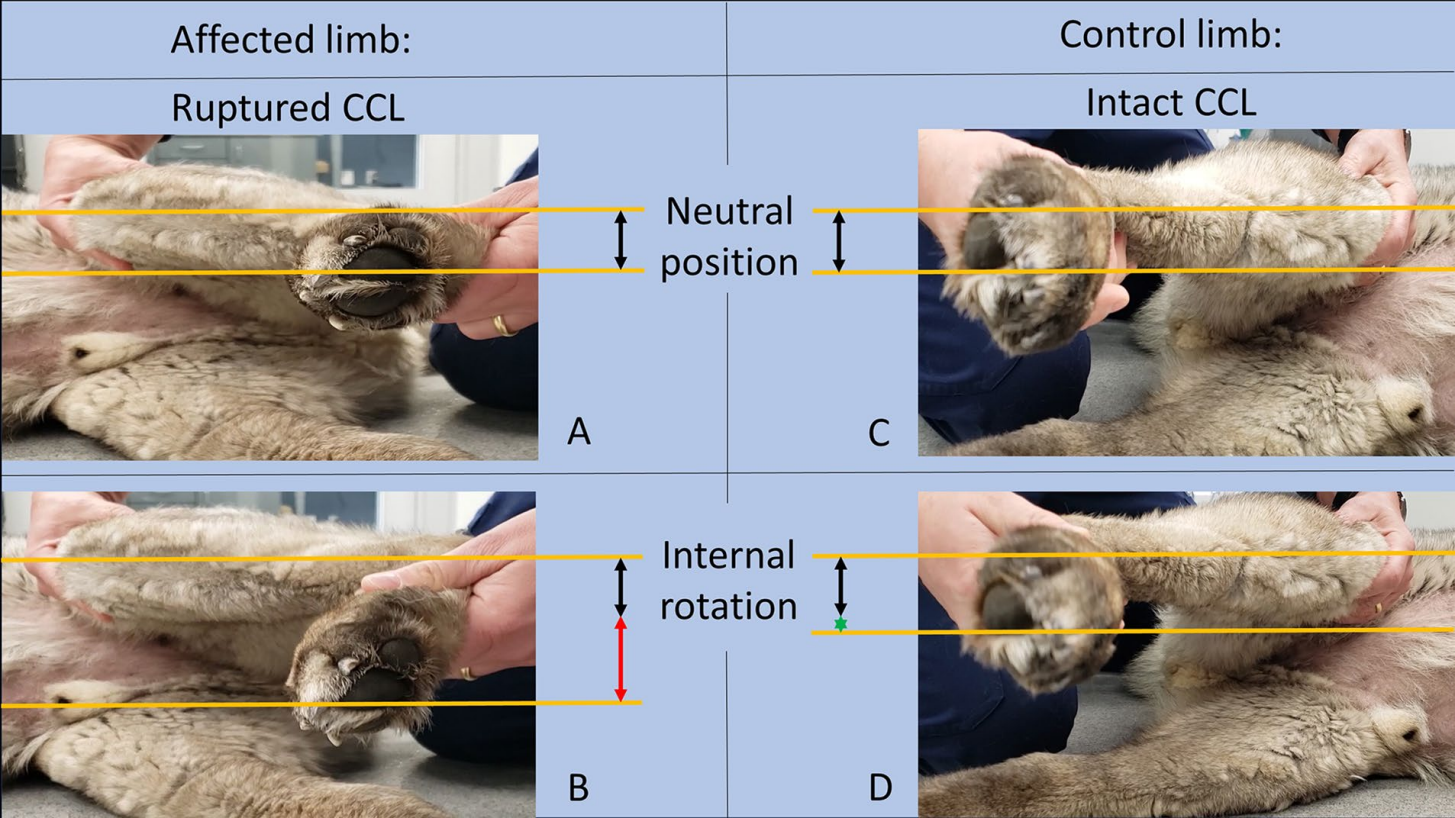
Images of the limb affected by CCLR on the left side **(A,B)** and the control limb with an intact CCL on the right side **(C,D)** in both a neutral position **(A,C)** and after RI-test-application **(B,D)**. The lines and arrows in the figure were added for illustrative purposes and were not utilized during clinical testing. The normal distance of the tibial tuberosity to the lower border of the fourth digital pad was indicated by black arrows. The greater rotational instability in the affected limb (**B**, red arrow) compared to the control limb (**C**, green asterisk).

To preoperatively identify “at-risk-dogs” for PS and aid in the decision regarding ARS-placement, the rotational-instability-test (RI-test) was applied bilaterally, and both in the awake and anesthetized dogs. During the RI-test, the limb was positioned at a standing stifle angle, with one hand fixed above the stifle and the other around the metatarsus. The latter was then gently rotated medially and movement of the tibial tubercle toward medially was observed (Figure 1; Supplementary video 1). Rotational instability was assigned a severity score based on visual assessment as follows: none to minimal (<30°, score = 0), mild (30–45°, score = 1), severe (>45°, score = 2). The rotational instability score (RI-score) was recorded. The intact contralateral stifle served as control. Actual rotational-instability-severity was used to determine whether or not an ARS was to be placed. It depended on the severity of rotational instability when compared to the intact side: a higher score in the CCL-deficient compared to the contralateral, intact, stifle, resulted in ARS placement. For instance, if a score of 0 was evident in the intact stifle, and a score of 1 or 2 in the affected stifle, an ARS was placed. Similarly, if a score of 1 was found in the intact stifle and a score of 2 in the affected, an ARS was also placed. If, however, mild rotational instability was noted on both sides (score of 1 bilaterally), then no ARS was placed.

### 2.3 Surgical technique

All surgeries obtained written owner consent and were conducted by a board-certified small animal surgeon (DVP; Small Animal Surgery Locum, PLLC) with 20 years’ experience in TPLO surgeries at study inception. Anesthetic protocols were standardized with regard to premedication, maintenance, administration of antibiotics and perioperative fluid therapy, and included femoro-sciatic nerve blocks prior to, and liposomal bupivacaine injections at completion of surgery. Owners could choose arthroscopic or mini-arthrotomy approaches to inspect the stifle joint. Intact menisci were released and torn menisci were partially resected using a #11-scalpel blade (mini-arthrotomy) or arthroscopic shaver/graspers (arthroscopy). The TPLO was performed as described (1), but without a jig (24) and without elevating the regional soft tissue envelope (25). Rotation was planned to achieve a post-operative tibial-plateau-angle of 1-3ᴼ. Included dogs received a 3.5 mm or broad 3.5 mm TPLO-plate (Veterinary Solutions Direct-Dover-Delaware-USA). This plate provides a slight (2-3 mm) space between its proximal end and the proximomedial tibia, facilitating ARS-anchoring.

**Figure 2:**
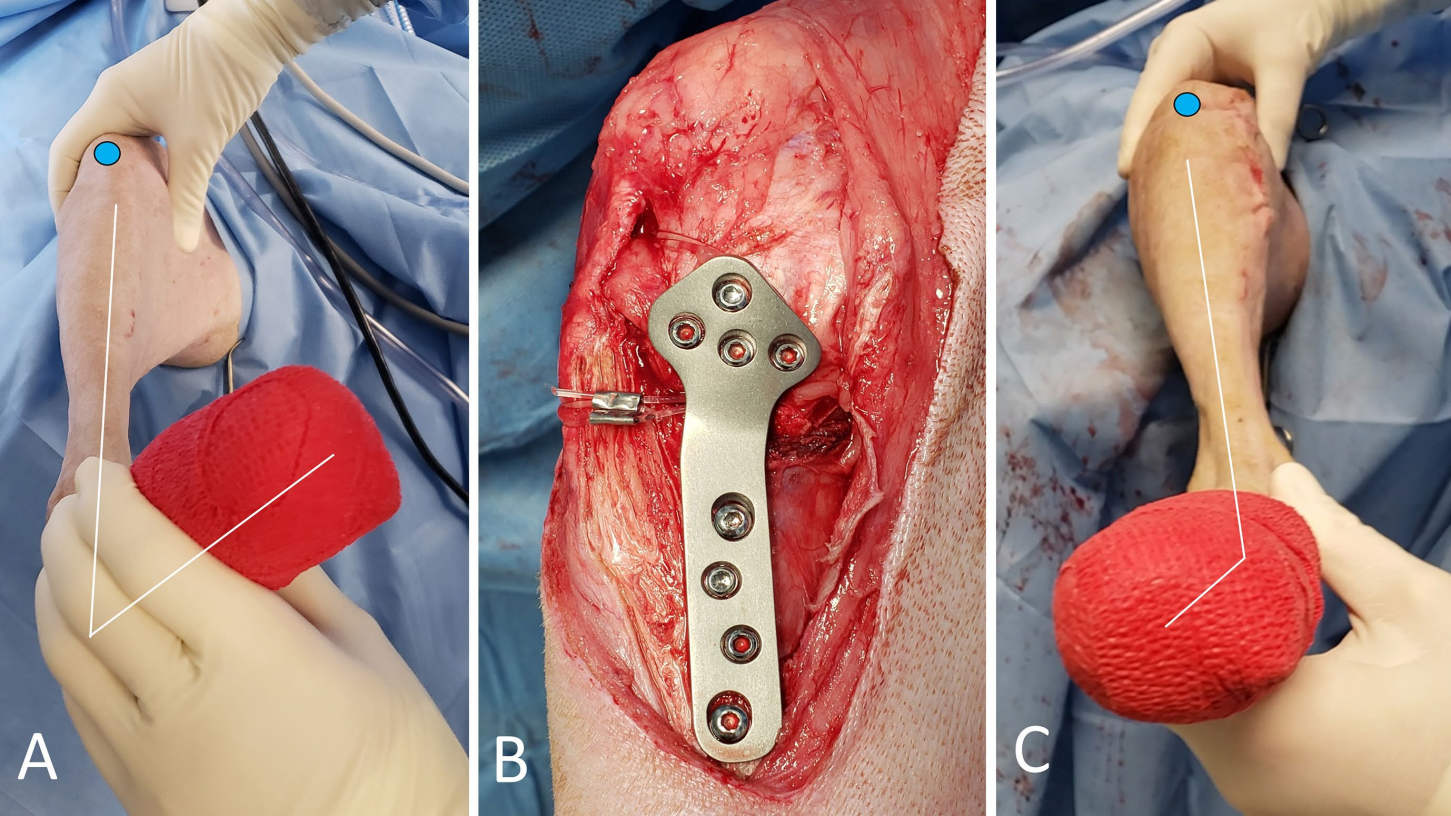
Photographs of the TPLO+ARS surgical procedure. The blue circle highlights the position of the patella. The RI-test is applied, revealing noticeable internal tibial rotation **(A)**. Appearance of the medial aspect of the stifle after completion of the TPLO with the ARS looped around the proximal aspect of the plate body, and secured **(B)**. The ARS effectively withstands the presurgically noted internal rotational instability (**C**, compare with image **A**).

**Figure 3:**
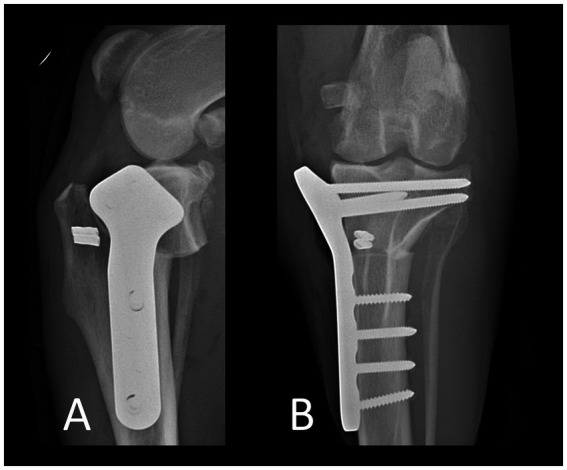
Orthogonal postoperative radiographs. Lateral view **(A)**. Craniocaudal view **(B)**.

The ARS consisted of a double-stranded monofilament nylon on a swaged-on needle (Securos Surgical-Fiskdale-Massachusetts-USA; sizes: 80 to 100-pound breaking strength). Prior to implantation, needle holders were placed at the ends of each strand, and the suture stretched as to remove memory. The strands were then placed around the lateral fabella. The distal two strands were tunneled below the tibialis cranialis muscle, emerging lateral to the proximal aspect of the tibial tuberosity and then guided around its cranial aspect. The proximal two strands were pulled caudal to the patellar ligament toward the medial tibial plateau. They were then either looped around the body of the TPLO-plate (below the most proximal 3 screws in a standard or below the most proximal 4 screws in a broad plate) or around the most proximocranial TPLO-screw, depending on which option allowed easier passage of the strands. After the ends of the corresponding proximal and distal strands were identified, a crimp clamp (Securos-Surgical-Fiskdale-Massachusetts-USA) was placed. An assistant then placed the limb in caudal drawer position with very slight external tibial rotation and the crimp clamp was secured. Prior to securing the crimps, the strands were not tightened with the same force as is typically done for a standard lateral fabellotibial suture. Instead, the tension was limited to a degree that avoided internal tibial rotation - in order to “catch” the rotation believed to occur during PS. However, due to its travel path, the ARS also reduced cranial tibial thrust. Following placement of the first crimp clamp, the stifle was assessed for cranial draw, tibial thrust and internal tibial rotation. Then, the stifle was held in a neutral position and the second strands were secured. The stifle was assessed for stability, and range of motion (Figure 2; Supplementary video 2), followed by lavage using sterile NaCl and standard closure in layers (0-PDS, 2–0 PDS, 3–0 Monocryl in a continuous pattern; application of tissue glue). Thereafter, RI-scores were recorded, a sterile dressing was applied, and orthogonal radiographs were taken (Figure 3).

### 2.4 Postoperative care

All dogs received cefpodoxime (5-10 mg/kg orally every 24 h), gabapentin (10 mg/kg orally every 8 h), carprofen (2.2 mg/kg orally every 12 h) for 14 days and trazodone (5 mg/kg orally every 8 h) as needed. Dressings were removed after 3 days. Elizabethan collars were mandatory to be worn until permission for removal from the surgeon, typically at 14 days, but only if the incision appeared completely healed.

A uniform postoperative activity protocol was applied. Indoors, dogs were limited to movement on carpeted areas on a single house level, without jumping on or off furniture. Stair-use was allowed only with guidance on the collar. Outdoor leashed walks started at 10 min for the first 2 weeks, then gradually increased to 45 min 3x/day until 8 weeks. If no gait abnormalities or any other clinical concerns were noted at 8 weeks, dogs could gradually resume normal activity over the following 4–6 weeks.

### 2.5 Follow-up

At 14 days postoperatively, owners had to send photographs of the incision and short videos for assessment. Owners were contacted to discuss progress and if no concerns were noted and the incision was healing normally, the Elizabethan collar could be removed and activity increased. If there were any concerns, owners were asked to present the dog for a direct examination.

A mandatory direct follow-up occurred at approximately 8 weeks. It involved a physical examination and radiographic evaluation by one of four board-certified surgeons with 4–20 years of TPLO-surgery-experience at study conduction. Standardized templates were employed for these examinations, covering lameness grade (26) and detection of abnormalities during visual and direct assessments, including evidence of PS during gait, testing for rotational instability, as well as assessment of radiographs. Without clinical or radiographic abnormalities, no further direct rechecks were needed. The direct follow-up period lasted until 8 weeks, unless complications arose, which were treated as needed and reported.

A final long-term follow-up phone call was conducted with all owners at a minimum of 12 months postoperatively to obtain any information regarding additional complications and concerns regarding the gait, specifically inquiring whether PS had been noted or not.

### 2.6 Radiographs

The tibial-plateau-angle was recorded pre-and postoperatively (1). Postoperative radiographs were obtained to also assess alignment, apposition and implant placement. On follow-up radiographs, the percentage of osseous osteotomy bridging was graded as follows: 0 = no bridging; 1 = 1–25%; 2 = 26–50%; 3 = 51–75%; 4 = 76–100% (27).

### 2.7 Complications and outcome

Major complications were defined as those needing medical treatment for more than 4 weeks or any additional surgical treatment; all other complications were considered minor (28). Surgical site infection (SSI) was defined as any irritation of the incision site, requiring topical or systemic antibiotic treatment or application of antiseptics to the incision. Outcomes were reported as full, acceptable or unacceptable (29).

### 2.8 Development of a novel TPLO plate design to simplify ARS anchorage

A TPLO-plate-design was conceptualized to facilitate the ARS-anchorage, avoiding positioning of the ARS around a screw or the TPLO-plate-body and eliminating the necessity to lift the plate away from the bone.

### 2.9 Statistical methods

Descriptive statistics were used for all of the continuous and ordinal factors. For assessment of any significant differences between the main groups (TPLO-only vs. TPLO+ARS), Chisquare or Fisher’s exact test were used. For nonnormal data sets, a Wilcoxon rank sum test (WRST) or Kruskal Wallis analysis was used. To assess if rotational instability was similar in the awake vs. the anesthetized patient, Chisquare analysis was used and to assess agreement, Kappa was calculated. For specific comparison for differences within this assessment, McNemar’s test was used. During comparisons between the subcategories of the two main groups (TPLO-only and mini-arthrotomy, TPLO-only and arthroscopy, TPLO+ARS and mini arthrotomy, TPLO+ARS and arthroscopy) and normal data, a one-way ANOVA was used, whereas for non-normal data, the Kruskal Wallis analysis was used. WRST was also used to compare whether or not anesthesia time, surgery time, or arthroscopy had a significant effect on complications (minor, major) and outcome (full, acceptable). Statistically significant difference was set as *p* ≤ 0.05. All analyses were performed with a statistical software program (SAS 9.3: PROC MIXED, PROC UNIVARIATE, PROC REG, v 9.3, SAS Institute, Cary, North Carolina).

## 3 Results

In total, 217 patients underwent TPLO-surgery during the study period. Of those, data of 132 dogs were omitted as those did not meet inclusion criteria (Table 1). Therefore, the final study cohort consisted of 85 dogs, which were, based on RI-test as reported above, grouped into TPLO-only (*n* = 57) and TPLO+ARS (*n* = 28).

**Table 1:** Reasons for exclusions.

Count	Reason for exclusion
44	Different plate sizes (none had ARS)^a^ 2.0 mm (12), 2.4 mm (5), 2.7 mm (12), 3.5 mm mini (14), 4.5 mm (2)
34	partially torn CCLR
21	MPL (4 of them with DFO)
3	no rechecks due to severe patient aggression
11	bilateral scopes, TPLO on one side only
2	Tibial angular limb deformity
17	Owner not reachable at long-term follow-up (but all underwent direct exam at 8 weeks without PS)
Total: 132	

a Anti-rotational suture.

The most common breed was the Labrador Retriever (*n* = 23), followed by Golden Retrievers (*n* = 11), Siberian Huskies (*n* = 8) and other breeds (*n* = 4 or less per breed); 43 dogs in total; Table 2.

**Table 2:** Listing of additional breeds, not named in the manuscript text.

Listing of additional included dog breeds (number of dogs)
German Shorthair Pointer (4), Pitbull Terrier (4), Cane Corso (4), Rottweiler (4), Mastiff (3), Border Collie (3), German Shepherd Mix (3), Springer Spaniel (2), Labradoodle (2), Great Pyrenees (2), 1 of each: English Bulldog, Boxer, Dogo Argentino, Catahoula Leopard dog, Fila Brasiliero, Hound, Great Dane, Collie, English Setter, Weimaraner, Akita, American Bulldog

Bloodwork results were unremarkable. Specific results on signalment, history, examination findings (including lameness grade (29) and rotational instability), surgery (including approach for joint inspection, meniscal status and treatment, as well as implants), radiographs, occurrence of PS, complications, follow-up and comparisons were summarized (Table 3; Supplementary Tables 1–4), with the most clinically relevant findings discussed as follows.

**Table 3:** Results and *p*-values from Wilcoxon rank sum test of factors comparing TPLO-only (*n* = 57) and TPLO+ARS (*n* = 28) cases.

Factor	Mean	SD^e^	SE^f^	Median	25th	75th	*p*
					Pctile^g^	Pctile^g^	
Weight (kg)^a^							
TPLO-only	35.70	8.54	1.13	36.2	28.8	43.0	0.99
TPLO+ARS	37.00	13.03	2.46	36.0	27.5	43.2	
Age (months)							
TPLO-only	77.42	28.54	3.78	76.0	52.0	100.0	0.67
TPLO+ARS	74.64	31.02	5.86	63.0	52.8	102.5	
Lameness duration (wks)^b^							
TPLO-only	14.41	23.93	3.17	4.0	2.0	17.0	0.14
TPLO+ARS	8.23	13.39	2.53	4.0	1.1	8.0	
Initial grade lameness							
TPLO-only	3.28	1.05	0.14	3.0	3.0	4.0	0.31
TPLO+ARS	3.50	0.92	0.17	4.0	3.0	4.0	
Pre-op TPA (ᴼ)^c^							
TPLO-only	30.15	2.98	0.39	31.0	27.0	33.0	0.2
TPLO+ARS	31.09	3.41	0.64	30.5	28.3	35.0	
Post-op TPA (ᴼ)^c^							
TPLO-only	2.68	1.90	0.25	2.2	1.4	3.7	0.75
TPLO+ARS	2.72	1.81	0.35	3.0	1.4	3.8	
Anesthesia time (min)^d^							
TPLO-only	149.39	40.90	5.42	152.0	132.5	170.5	0.01
TPLO+ARS	180.82	44.81	8.47	175.0	146.0	196.0	
Surgery time (min)^d^							
TPLO-only	58.60	14.54	1.93	58.0	46.5	69.5	< 0.001
TPLO+ARS	77.50	19.20	3.63	76.5	65.3	91.0	
1st radiographic recheck (wks)^b^							
TPLO-only	8.74	1.33	0.18	8.0	8.0	9.0	0.6
TPLO+ARS	8.96	1.45	0.27	9.0	8.0	9.0	
Bone healing on radiographs							
TPLO-only	3.71	0.46	0.06	4.0	3.0	4.0	0.03
TPLO+ARS	3.89	0.42	0.08	4.0	4.0	4.0	
8-wks^b^ grade lameness							
TPLO-only	0.71	0.84	0.11	0.5	0.0	1.0	0.62
TPLO+ARS	0.56	0.66	0.13	0.5	0.0	1.0	
Last phone call (days)^b^							
TPLO-only	628.70	170.85	22.63	648.0	511.0	799.5	0.1
TPLO+ARS	578.93	175.20	35.57	616.5	360.3	751.3	

a kilogram.

b weeks.

c preoperative and postoperative tibial-plateau-angle.

d minutes.

e standard deviation.

f standard error of the mean.

g percentile.

Dogs receiving an ARS had a mean lameness duration prior to surgery of approximately 8 weeks, while dogs in the TPLO-only group were roughly 14.5 weeks lame, without a statistically significant difference (*p* = 0.14). Similarly, when comparing the various parameters between groups (Table 3), no significant differences could be detected with the exception of the following: mean surgery time was approximately 20 min longer (*p* = 0.01) and anesthesia time was approximately 30 min longer (*p*<0.001) when an ARS was placed. In those dogs, the mean bone healing scores at the 8-week recheck was also significantly higher (*p* = 0.03).

When assessing associations between the evaluated factors and whether or not an ARS was placed, no significant differences were found, other than those used to determine appropriateness for ARS, i.e., assessment of rotational instability in the awake patient, under anesthesia and when assessing the score of rotational instability (<0.001 each) (Supplementary Table 1). While not showing rotational instability in the awake patient, 7 cases were found to have rotational instability when anesthetized. Statistical agreement calculations (Kappa = 0.69 and McNemar’s test *p* = 0.065) revealed that an examiner can be 93.5% confident that there is a difference when comparing rotational instability prior to and after anesthesia. The RI-score immediately after TPLO-ARS surgery was 0 in all dogs of that group and remained unchanged to pre-surgical values when recorded under anesthesia in TPLO-only dogs.

At the 8-week-recheck, PS was noted in 2/57 dogs in the TPLO-only group. The RI-score in both PS-positive dogs had increased from 0 immediate post-operatively to 1 at that recheck. No score-change was noted in the remaining 55 dogs. Therefore, the RI-test had failed to reliable identify dogs at risk to develop PS. Conversely, at 8 weeks, 0/28 in the TPLO+ARS group (*p* = 1.00) showed PS, with no dog showing rotational instability (RI-scores = 0).

The owners of the PS-positive dogs in the TPLO-only group declined an additional surgery to place an ARS. According to later follow-up via telephone with those owners, PS disappeared gradually in both dogs over the following 3–6 months.

Minor complications developed in 6 dogs of the TPLO-only group and 2 dogs of the TPLO+ARS group. Those 8 cases reflect a minor complication rate of 9.4%. All were mild superficial irritations of the surgical site and in all cases, the owners had removed the Elizabethan collar within a few days post-surgery, contrary to the specific discharge instructions. Treatment with systemic antibiotics (cefpodoxime; 5-10 mg/kg orally every 24 h) for 5–10 days, locally applied mupirocin ointment and extended wearing of the Elizabethan collar resolved those concerns in all 8 dogs initially, but 3 of them showed recurrence of infection (2 in the TPLO-only group, 1 in the TPLO+ARS group).

Major complications consisted of implant removal in 3 dogs (3.5% major complications). Two dogs of the TPLO-only-group developed recurring mild draining tracts between 3 and 6 months post-operatively. Implant explantation was performed in both dogs at 4 and 7 months, respectively. The other major complication occurred in 1 TPLO+ARS-dog. This dog showed sudden onset of grade 2/5 lameness and evidence of PS immediately after owners allowed the dog to run freely in a large field, a few days following the 8-week-recheck, when the dog had been cleared to slowly be reconditioned over the following 4–6 weeks. Examination at 11 weeks postoperatively revealed the RI-score had increased from 0 (immediate post-surgery) to 2 (at 11 weeks). In addition, a superficial infection progressed despite antibiotics over the following 5 weeks, requiring implant removal at 16 weeks after the original surgery. At that time, while the crimp clamps were found to have remained secure, the ARS-strands had become stretched and loose, no longer preventing internal rotation – likely the reason for PS in this dog. All dogs recovered well without evidence of PS-recurrence.

Meniscal injury, treatment or release were not associated with development of PS (*p* = 1.00, *p* = 0.58, *p* = 0.58, respectively). However, since this study did not exclusively evaluate dogs that underwent TPLO without ARS, and given the possibility that ARS placement may have prevented PS in dogs with meniscal injury, the current study design does not permit conclusions regarding a potential correlation between meniscal injury and the development of PS.

None of the other examined factors showed an effect on PS-development (Supplementary Tables 2, 3), including no difference between whether a recheck was seen by the board-certified surgeon who operated on the dog or another board-certified surgeon. This statistical finding excluded influence of surgeon-bias on outcome.

No difference was found when assessing if anesthesia time, surgery time, arthroscopy, ARS-placement, or plate type (standard vs. broad) had a significant effect on complications (minor, major) and outcome (full, acceptable) (Supplementary Table 4).

There was no difference in time of final follow-up telephone call (630 and 580 days postoperatively in both groups; Table 1, *p* = 0.1). All dogs were graded as having full outcome and none of the owners noted any gait abnormality at final follow-up.

**Figure 4:**
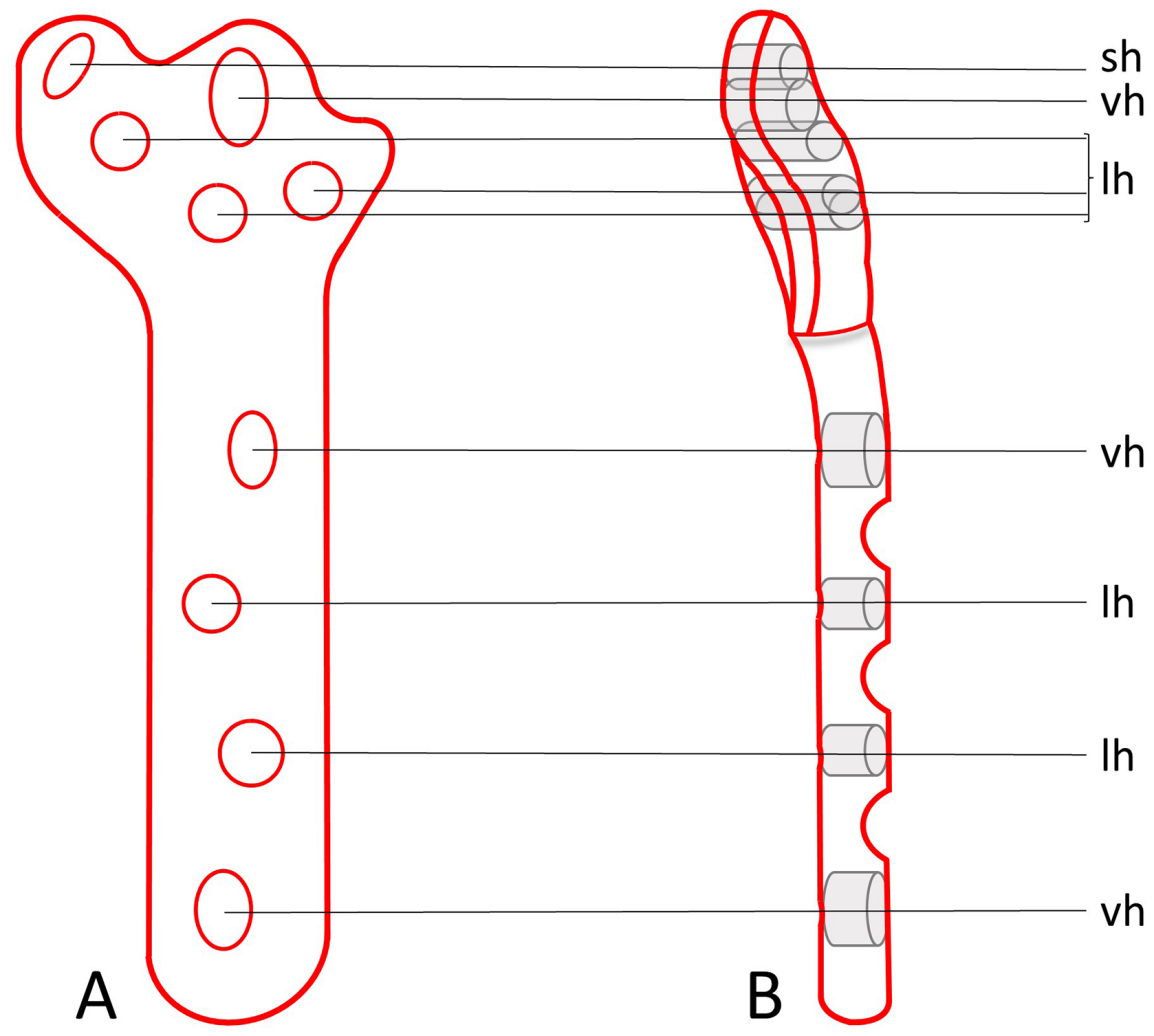
Orthogonal illustrations **(A,B)** of the envisioned prototype for a TPLO/ARS-plate. The design includes an extension of the plate material at the cranioproximal plate border to accommodate the addition of a hole to anchor the ARS (sh). The remaining plate-holes function for the placement of screws at variable angles (vh) or serve as locking holes (lh).

**Figure 5:**
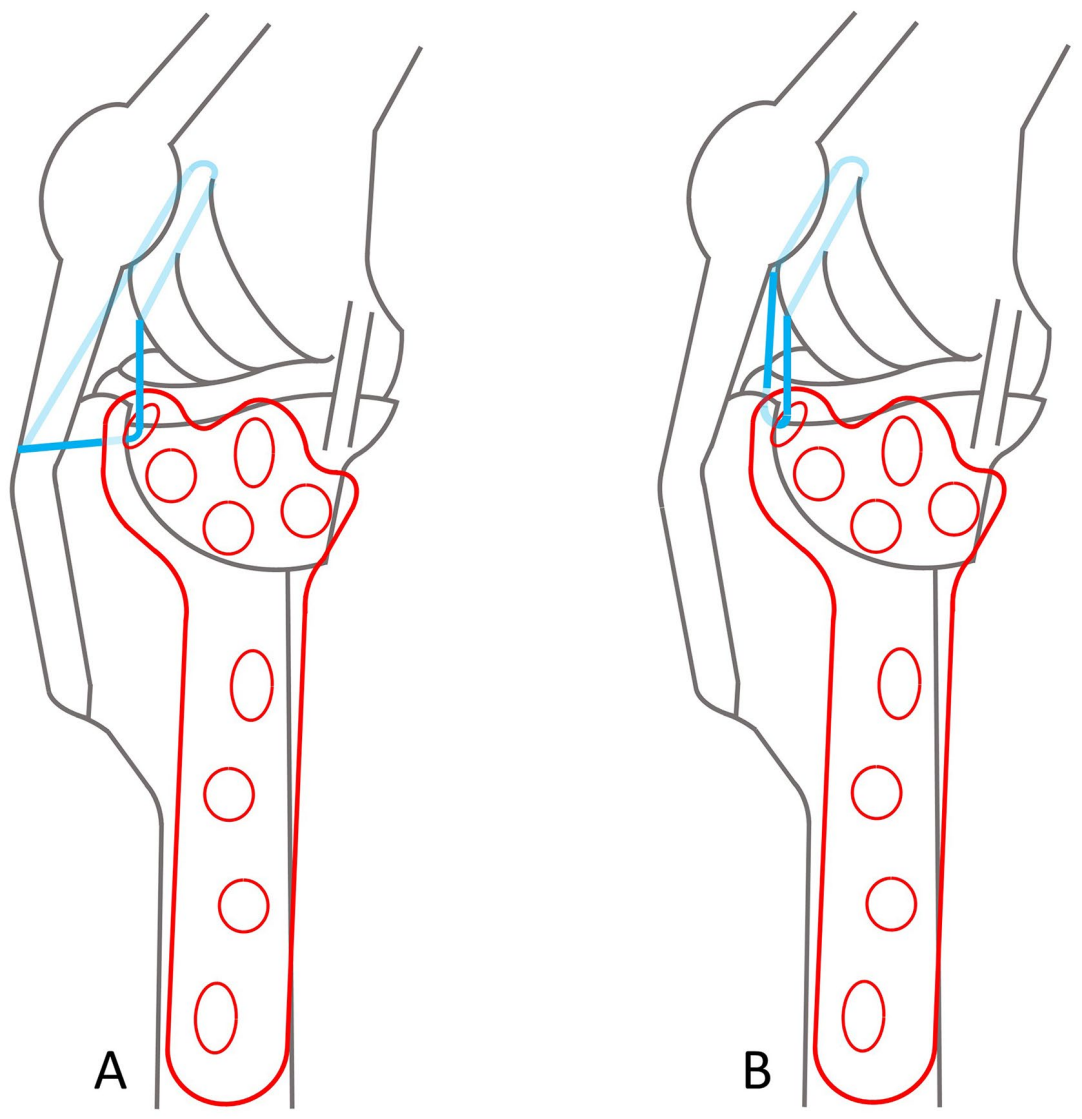
Diagrams of a TPLO/ARS plate, illustrating options for the ARS-travel path. In both scenarios, the ARS is medially anchored through the ARS-anchoring-hole, and around the lateral fabella. Dark blue lines depict visible suture, transparent blue indicates ARS-suture traveling around the plate or anatomical structures that would otherwise not allow visualization. **(A)** The proximal strand is positioned caudal to the patellar ligament and the distal strand encircles the proximal aspect of the tibial tuberosity. **(B)** Both strands travel caudal to the patellar ligament.

The design process of the TPLO/ARS-plate underwent a comprehensive engineering procedure, the full details of which are omitted in this manuscript for brevity. In short, similar to the presently available anatomically shaped precontoured TPLO-plates, the novel TPLO/ARS-plate maintains contouring to match the medial aspect of the proximal tibia and incorporates a limited-contact shaft to minimize plate-to-bone contact area, promoting vascularity preservation to aid with bone healing. The novel design involved extending the cranioproximal aspect of the plate to provide additional material through which a hole can be placed, facilitating ARS-anchoring (suture hole; Figure 4). This modification allows for ARS-placement as performed in the current study (with the proximal strand positioned caudal to the patellar ligament and the distal strand encircling the proximal aspect of the tibial tuberosity), or with both strands traveling caudal to the patellar ligament (Figure 5).

## 4 Discussion

Based on the RI-test results, 57 dogs did not receive an ARS, but as 2 of these exhibited PS, the first hypothesis was rejected. No TPLO+ARS dog developed PS in the first 8 weeks and while 1 TPLO+ARS dog developed PS at 11 weeks due to premature activity and ARS-stretching, the second hypothesis was accepted. The third hypothesis was also accepted: independent of group, all dogs returned to normal gait without PS. A novel TPLO-plate was designed, with potential for future applications.

Our study aimed to show no significant differences between groups. This goal was largely achieved, with a well-balanced distribution of various factors between groups. As explained in the introduction, to achieve sufficient power, two groups of over 250 dogs would have been needed. As it did not seem ethical to perform a TPLO+ARS to that many dogs without any prior pilot data, we opted to collect such needed pilot data first. Following data collection, and regardless of the small PS-numbers, statistical analyses were still conducted to potentially extract valuable information such as identification of factors predisposing dogs to develop PS. Regardless, while the data collected in this pilot study can be used as the base for those future investigations, our statistical results have therefore to be cautiously interpreted.

Owing to the additional procedural steps required with TPLO+ARS, this technique demonstrated expectedly approximately 20 min and 30 min longer anesthesia and surgery times. Considering the lack of differences in complications between the groups, accepting these extended times appears justified in exchange for a reduced risk of PS-development.

The higher radiographic bone healing scores following TPLO+ARS (Table 3) are not fully understood. One could speculate that the additional dissection for ARS-placement may result in mild regional vascular damage with subsequent formation of local hematoma, that could provide an increased supply of growth factors, which in turn might have enhanced bone healing, but this is speculative at this time.

We could not confirm results of previous work that suggested meniscal injury or meniscal treatment increases cranial tibial subluxation and thus PS (3, 6). Similar to those reports, our case numbers were limited and therefore, the effect from meniscal injury or -treatment on PS warrants further investigation. Future studies with larger case numbers might reveal more information on the association between meniscal treatment and PS-development. A meniscal release destructs a structure that contributes to stifle stability and performing different meniscal treatments is likely a confounding factor in the current study’s statistical analysis.

Similar to others (3), our mean pre-and postoperative TPAs were not identified as risk factors for PS. In our study, rotation of the tibial plateau was aimed to be approximately 1-3ᴼ, believed to reduce remaining post-operative cranial tibial thrust (8, 30, 31).

Our cases involved dogs with prolonged lameness, contrasting with a study that restricted ARS-placement to dogs with acute CCLR and hyperlax stifles (15). In those dogs, the absence of periarticular fibrosis has been suggested to contribute to PS (3, 15, 16), whereas its presence has been reported to reduce overall instability (32). This might explain, why TPLO-only-dogs might have had lower rotational instability scores compared to TPLO+ARS-dogs. However, PS has also been observed up to 1-year post-TPLO (10), a period when a large amount of periarticular fibrosis is expected. The role and extent of periarticular fibrosis in the etiology of PS remains unclear, evidenced also by contradictory reports where satisfactory PS-resolution was achieved conservatively in one report (2), while only 25% improvement was reported in another study (3).

We compared CCLR-affected and non-affected stifles with RI-scores. Each patient served as their own control, enabling a personalized assessment of normal versus increased instability. A grading system was employed, deemed practical for routine use in the clinical, in-vivo, setting. We assert that the adopted scoring system, along with its defined ranges of instability, should be easily reproducible by others. This stands in contrast to a recent cadaveric in-vitro study, employing three-dimensional tracking camera systems and goniometric measurements (16). While such apparatus employed in a laboratory university setting undoubtedly offers precise measurements by providing objective data, its implementation in a bustling surgical practice would, in the humble opinion of the authors of the current study, prove impractical. However, in hindsight, the use of smartphone apps and/or plastic goniometers to more precisely measure rotational instability could have been useful to obtain stronger data.

As stated in the introduction, the RI-test was employed based on our clinical experience over the past decade and the observation that dogs presenting with post-TPLO-PS typically exhibited high RI-scores. However, the RI-test has not been formally validated. Consequently, although unlikely, it is possible that its inaccuracy may have led to failure in identifying dogs predisposed to post-TPLO-PS or to the unnecessary placement of an ARS in dogs that may not have required it. Despite these limitations, surgeons must weigh the risk of a dog developing post-TPLO-PS and potentially requiring revision surgery against the relatively minor additional time - approximately 20 min - needed to perform an ARS in dogs considered at high risk. Further studies are warranted to address this question. Validation of the RI-test and definitive conclusions regarding its utility were beyond the scope of this pilot study. Due to the lack of validation of the RI-test, it can only be concluded that ARS-placement might help prevent PS; however, it remains unknown how many of the dogs in the TPLO+ARS-group would have developed PS in the absence of ARS-placement.

Our results indicate that RI-scores recorded with dogs under anesthesia were more reliable indicators for ARS placement decision-making. Most veterinarians would likely agree that certain dogs pose greater challenges during examination while conscious. Consequently, the RI-test was administered on both conscious and anesthetized patients. This approach aimed to mitigate the risk of overlooking rotational instability in awake dogs. On the other hand, examining dogs under anesthesia presents a potential drawback due to diminished muscle tone, possibly leading to the observation of non-clinically relevant instability. Importantly, two previous studies assessing dogs for predisposition to PS (16, 33), including the tibial-pivot-compression-test (33), published subsequent to our study, reportedly utilized cadaveric specimens. Assessing the risk for PS in anesthetized patients or cadaveric specimens with a lack of muscle tone might carry the risk of over diagnosing a problem. The sensitivity of any test to identify dogs predisposed to PS in awake and anesthetized patients must be proven in future studies.

Two TPLO-only dogs developed PS, suggesting that there is a lack of sensitivity of the applied RI-test. Therefore, additional assessment of the RI-test’s ability to reliably predict PS occurrence post-TPLO is indicated, with focus on sensitivity and specificity along with information about the degree of inter-operator variations. In addition, as the 3 dogs experiencing PS post-surgery recovered without additional intervention, the necessity for initial TPLO+ARS surgery should be considered. One could argue that ARS-placement may be warranted solely for cases developing PS post-TPLO and not responding to an initial conservative approach. Conversely, it could be argued that ARS-incorporation in dogs that seem to be predisposed prevents the occurrence of PS from the outset, justifying the slightly increased surgery time and associated costs. Indeed, anecdotally, some surgeons apply an ARS routinely to TPLOs.

Regardless, if a procedure to prevent PS is indicated, then the most appropriate technique and implant material should be used. The “internal brace” technique states it involves bone anchor-placement at isometric points (16, 34). As the exact location of true isometric points remains speculative and likely varies throughout joint range of motion (35, 36), and the ARS aims to mitigate internal tibial rotation, with reduced strain compared to a true fabellotibial suture due to reduced cranial tibial thrust post-TPLO, the need for isometric points may be less crucial (6, 15, 36). While bone anchors showed biomechanical superiority over securing the suture around the lateral fabella in-vitro (37), in-vivo comparisons remain unexplored. Risks of anchor pull-out and suture-breakage exist (20) and current studies on anchor-use with the “internal brace” are limited to in-vitro investigations (38).

While utilizing UHMWPE alone for stabilizing a cranial cruciate ligament (CCL)-deficient stifle may pose an elevated risk of complications, specifically infection (5), it performs superior to monofilament suture when biomechanically tested in cadaveric specimens (37). Moreover, the stability of a suture anchored to the lateral fabella relies on the strength of the fabellofemoral soft tissue attachment, and instability in that area might contribute to complications including meniscal injury or persistent/recurrent stifle instability (39). In contrast, the forces exerted on an adjunctive ARS when employed alongside TPLO are likely considerably reduced compared to using a suture as the sole means of stabilizing a CCL-deficient stifle. Consequently, the concerns regarding the aforementioned complications are likely mitigated with TPLO+ARS, as utilized in the current study. Biomechanical ex-vivo and prospective, comparative in-vivo studies would be necessary to delineate any disparities between TPLO+ARS, as outlined in our study, and the “internal brace” method.

In contrast to securing the suture through a tibial bone tunnel as with the “internal brace,” the proposed TPLO/ARS-plate may present an alternative option for ARS anchoring. While bearing similarities to other plates (DePuy Synthes-Zuchwill-Switzerland; Arthrex-Naples-Florida-USA), our novel design distinguishes itself as described above and shown in Figure 4. The main distinction compared to other plates is that no additional instrumentation is required, and a monofilament suture can be used instead of a braided one as with the internal brace. These features may lower costs for clients (approximately $210 for the former: Securos Surgical, Tuttlingen, Germany, February 09, 2022, personal communication, compared to approximately $1850 for the latter, only counting implant material while excluding costs for special equipment needed: Arthrex Swiss AG, Belp, Kanton Bern August 18, 2023, personal communication) and, based on previous observations (5), could also reduce the risk of infection (5). The objective for the proposed plate-design stemmed from the acknowledgment that the technique for ARS-placement, as executed in our cases, presents potential technical challenges such as placement around the plate if the latter is too close to the bone or if the screw shaft is not exposed sufficiently. One particular concern is securely fastening the ARS-suture around the most proximocranial screw shaft. This method carries the inherent risk of the monofilament suture being susceptible to abrasion over time from the screw threads. Consequently, a central objective throughout our study was to devote considerable dedication to invest time and resources from conceptualization to finalizing technical drawings of the novel TPLO/ARS-plate design. This design has not been submitted for patent application to prevent financial or inventor bias. Instead, it is openly shared with the aspiration that it can be embraced by others, including manufacturers and patent-holders of previously designed plates. It is of note that it is not the intention of the authors to infringe on the patent of that previously reported plate (40). The authors declare no financial interest in the proposed plate design. Production, in vitro and in vivo testing of the novel plate are indicated to ensure this implant is of comparable strength and performance to currently available implants. The arduous and time-consuming production and testing of this implant was beyond the scope and timeframe of this study.

Whether the ARS is positioned similar to the current study or with both strands traveling caudal to the patellar ligament is a subject for future investigation (Figure 5). The latter option might mitigate the risk of potential frictional irritation of the tibial tuberosity. In line with the development of the proposed TPLO/ARS-plate, the authors deem it imperative to remain cognizant of these potential clinical applications for future testing, with the aim of minimizing potential harm for dogs undergoing such procedures in the future, although we believe the risk for complications is low, provided proper antiseptic and surgical technique is applied.

When a TPLO is performed by an experienced surgeon, major and minor complication rates are reported as 3.1 and 8.3% (4). Those numbers are similar to our 3.5 and 9.4%. While, based on the results from this study, we can conclude that TPLO+ARS does not seem to carry an increased risk for complications, future cases might reveal additional complications.

Limitations of this study include the low case numbers, due to its pilot nature and purposefully deciding to proceed as explained above. Grouping was not randomized to avoid assigning a dog with severe rotational instability to receive only a TPLO, potentially predisposing it for PS-development. While surgeon-bias was excluded, not one of the surgeons was blinded to the treatment and follow-up data was obtained subjectively. Force plate analysis and three-dimensional stifle motion analyses could be topic for future research.

In conclusion, the TPLO+ARS procedure appears to be a feasible surgical technique. While acknowledging that the RI-test used in this study is not validated - and thus cannot reliably determine whether ARS placement is indicated - ARS may still be considered as a potential method to reduce the incidence of post-TPLO-PS, given ease of application with readily available implant material, and minimal additional surgical time. The proposed TPLO/ARS plate design requires further biomechanical and clinical evaluation.

## Data Availability

The original contributions presented in the study are included in the article/Supplementary material, further inquiries can be directed to the corresponding author.
