# Supplementary material for: Monofilament anti-rotational suture combined with TPLO to prevent pivot shift: surgical technique and novel TPLO plate design
Source: Front Vet Sci. 2025 Sep 18;12:1456869. doi: 10.3389/fvets.2025.1456869 (PMC12490421; doi:10.3389/fvets.2025.1456869)
Supplement: Supplementary file 1 [file Data_Sheet_1.docx]

Supplementary Table 1a: Results and P-values from Chi-Square or Fisher's exact test of factors comparing TPLO-only (n=57) and TPLO+ARS (n=28) cases and showing associations.

| **Factor** | **TPLO-only** | **TPLO+ ARS** | **Total** | **P** |
| --- | --- | --- | --- | --- |
| **Sex** |  | | | |
| F^a^ | 1 | 0 | 1 | 0.67 |
| FS^b^ | 30 | 15 | 45 |  |
| M^c^ | 2 | 0 | 2 |  |
| MN^d^ | 24 | 13 | 37 |  |
| **Rotational instability awake** |  | | | |
| No | 55 | 9 | 64 | ˂ 0.001 |
| Yes | 2 | 19 | 21 |  |
| **Rotational instability anesthetized** |  | | | |
| No | 57 | 0 | 57 | ˂ 0.001 |
| Yes | 0 | 28 | 28 |  |
| **Rotational instability score anesthetized** |  | | | |
| 0 | 51 | 0 | 51 | ˂ 0.001 |
| 1 | 5 | 6 | 11 |  |
| 2 | 1 | 22 | 23 |  |
| **Side** |  | | | |
| Left | 31 | 15 | 46 | 1.00 |
| Right | 26 | 13 | 39 |  |
| **Meniscal injury** |  | | | |
| No | 24 | 14 | 38 | 0.64 |
| Yes | 33 | 14 | 47 |  |
| **Partial meniscectomy** |  | | | |
| No | 29 | 18 | 47 | 0.26 |
| Yes | 28 | 10 | 38 |  |
| **Meniscal release** |  | | | |
| No | 28 | 10 | 38 | 0.26 |
| Yes | 29 | 18 | 47 |  |
| **Plate Type** |  | | | |
| 3.5mm standard | 38 | 17 | 55 | 0.63 |
| 3.5mm broad | 19 | 11 | 30 |  |
| **SSI**^e^ |  | | | |
| No | 50 | 25 | 75 | 1.00 |
| Yes | 7 | 3 | 10 |  |

^a^female, ^b^female/spayed, ^c^male, ^d^male/neutered, ^e^surgical site infection.

Supplementary Table 1b: Results and P-values from Chi-Square or Fisher's exact test of factors comparing TPLO-only (n=57) and TPLO+ARS (n=28) cases and showing associations.

| **Factor** | **TPLO-only** | **TPLO+ ARS** | **Total** | **P** |
| --- | --- | --- | --- | --- |
| **Major complication** |  | | | |
| No | 56 | 26 | 82 | 1.00 |
| Yes | 1 | 2 | 3 |  |
| **Minor complication** |  | | | |
| No | 51 | 26 | 77 | 0.42 |
| Yes | 6 | 2 | 8 |  |
| **Pivot Shift (8 weeks)** |  | | | |
| No | 55 | 28 | 83 | 1.00 |
| Yes | 2 | 0 | 2 |  |

Supplementary Table 2a: Results and P-values from Wilcoxon rank sum test of factors comparing whether no pivot shift (PS) occurred (n=82) or whether it did occur (n=3), depending on various factors.

| **Factor and PS (no or yes)** |  |  |  |  | **25th** | **75th** |  |
| --- | --- | --- | --- | --- | --- | --- | --- |
|  | **Mean** | **SD**^e^ | **SE**^f^ | **Median** | **Pctile**^g^ | **Pctil**^g^ | **P** |
| **Weight (kg)**^a^ |  | | | | | | |
| No | 36.27 | 10.32 | 1.14 | 36.1 | 27.8 | 43.1 | 0.47 |
| Yes | 32.09 | 3.24 | 1.87 | 30.5 | 30.0 | 35.8 |  |
| **Age (months)** |  | | | | | | |
| No | 77.00 | 29.12 | 3.22 | 75.5 | 52.0 | 100.0 | 0.46 |
| Yes | 63.00 | 35.38 | 20.43 | 57.0 | 31.0 | 101.0 |  |
| **Lameness duration (wks)**^b^ |  | | | | | | |
| No | 12.24 | 21.42 | 2.37 | 4.0 | 2.0 | 12.3 | 0.23 |
| Yes | 16.00 | 14.42 | 8.33 | 12.0 | 4.0 | 32.0 |  |
| **Initial grade lameness** |  | | | | | | |
| No | 3.37 | 0.99 | 0.11 | 3.5 | 3.0 | 4.0 | 0.88 |
| Yes | 3.00 | 1.73 | 1.00 | 4.0 | 1.0 | 4.0 |  |
| **Pre-op TPA**^c^ **(ᴼ)** |  | | | | | | |
| No | 30.49 | 3.10 | 0.34 | 31.0 | 27.8 | 33.0 | 0.64 |
| Yes | 29.67 | 4.73 | 2.73 | 28.0 | 26.0 | 35.0 |  |
| **Post-op TPA**^d^ **(ᴼ)** |  | | | | | | |
| No | 2.62 | 1.75 | 0.19 | 2.4 | 1.4 | 3.7 | 0.45 |
| Yes | 4.50 | 3.97 | 2.29 | 3.0 | 1.5 | 9.0 |  |
| **Anesthesia time (min)**^d^ |  | | | | | | |
| No | 159.21 | 45.03 | 4.97 | 156.0 | 138.8 | 178.3 | 0.36 |
| Yes | 174.33 | 27.68 | 15.98 | 178.0 | 145.0 | 200.0 |  |
| **Surgery time (min)**^d^ |  | | | | | | |
| No | 64.68 | 18.41 | 2.03 | 64.5 | 52.0 | 76.0 | 0.86 |
| Yes | 68.67 | 22.30 | 12.88 | 60.0 | 52.0 | 94.0 |  |

^a^kilogram, ^b^weeks, ^c^preoperative and ^d^postoperative tibial-plateau-angle, ^d^minutes, ^e^standard deviation, ^f^standard error of the mean, ^g^percentile.

Supplementary Table 2b: Results and P-values from Wilcoxon rank sum test of factors comparing whether no pivot shift occurred (n=82) or whether it did occur (n=3), depending on various factors.

| **Factor and PS (no or yes)** |  |  |  |  | **25th** | **75th** |  |
| --- | --- | --- | --- | --- | --- | --- | --- |
|  | **Mean** | **SD**^c^ | **SE**^d^ | **Median** | **Pctile**^e^ | **Pctil**^e^ | **P** |
| **1st radiographic rechck (wks)**^a^ |  | | | | | | |
| No | 8.81 | 1.36 | 0.15 | 9.0 | 8.0 | 9.0 | 0.85 |
| Yes | 9.00 | 2.00 | 1.15 | 9.0 | 7.0 | 11.0 |  |
| **8-wks**^a^ **grade lameness** |  | | | | | | |
| No | 0.64 | 0.77 | 0.09 | 0.5 | 0.0 | 1.0 | 0.26 |
| Yes | 1.33 | 1.15 | 0.67 | 2.0 | 0.0 | 2.0 |  |

^a^weeks, ^c^standard deviation, ^d^standard error of the mean, ^e^percentile.

Supplementary Table 3a: Results and P-values from Chi-Square or Fisher's exact test of factors comparing whether no pivot shift occurred (n=82) or whether it did occur (n=3) and showing associations.

| **Factor** | **Pivot shift** | |  |  |
| --- | --- | --- | --- | --- |
|  | **No** | **Yes** | **Total** | **P** |
| **Sex** |  | | | |
| F^a^ | 1 | 0 | 1 | 0.26 |
| FS^b^ | 45 | 0 | 45 |  |
| M^c^ | 2 | 0 | 2 |  |
| MN^d^ | 34 | 3 | 37 |  |
| **Rotational instability awake** |  | | | |
| No | 62 | 2 | 64 | 1.00 |
| Yes | 20 | 1 | 21 |  |
| **Rotational instability anesthetized** |  | | | |
| No | 55 | 2 | 57 | 1.00 |
| Yes | 27 | 1 | 28 |  |
| **Rotational instability score anesthetized** |  | | | |
| 0 | 49 | 2 | 51 |  |
| 1 | 11 | 0 | 11 | 0.79 |
| 2 | 22 | 1 | 23 |  |
| **Side** |  | | | |
| Left | 44 | 2 | 46 | 1.00 |
| Right | 38 | 1 | 39 |  |
| **Meniscal injury** |  | | | |
| No | 37 | 1 | 38 | 1.00 |
| Yes | 45 | 2 | 47 |  |
| **Partial meniscectomy** |  | | | |
| No | 46 | 1 | 47 | 0.58 |
| Yes | 36 | 2 | 38 |  |
| **Meniscal release** |  | | | |
| No | 36 | 2 | 38 | 0.58 |
| Yes | 46 | 1 | 47 |  |
| **Plate Type** |  | | | |
| 3.5mm standard | 53 | 2 | 55 | 1.00 |
| 3.5mm broad | 29 | 1 | 30 |  |

^a^female, ^b^female/spayed, ^c^male, ^d^male/neutered.

Supplementary Table 3b: Results and P-values from Chi-Square or Fisher's exact test of factors comparing whether no pivot shift occurred (n=82) or whether it did occur (n=3) and showing associations.

| **Factor** | **Pivot shift** | |  |  |
| --- | --- | --- | --- | --- |
|  | **No** | **Yes** | **Total** | **P** |
| **Joint inspection type +/- ARS** |  | | | |
| TPLO + mini approach | 20 | 0 | 20 | 0.63 |
| TPLO + arthroscopy | 35 | 2 | 37 |  |
| TPLO + mini approach + ARS | 10 | 0 | 10 |  |
| TPLO + arthroscopy + ARS | 17 | 1 | 18 |  |
| **Recheck (TPLO-only or TPLO+ARS)** |  | | | |
| **by original surgeon** |  |  |  |  |
| No | 42 | 2 | 44 | 1.00 |
| Yes | 40 | 1 | 41 |  |
| **Bone healing on radiographs** |  | | | |
| 0 | 0 | 0 | 0 | 0.8 |
| 1 | 0 | 0 | 0 |  |
| 2 | 1 | 0 | 1 |  |
| 3 | 16 | 1 | 17 |  |
| 4 | 65 | 2 | 67 |  |
| 5 | 0 | 0 | 0 |  |
| **SSI**^a^ |  |  |  |  |
| No | 72 | 2 | 75 | 1.00 |
| Yes | 10 | 1 | 10 |  |
| **Major complication** |  | | | |
| No | 80 | 2 | 82 | 1.00 |
| Yes | 2 | 1 | 3 |  |
| **Minor complication** |  | | | |
| No | 75 | 2 | 77 | 1.00 |
| Yes | 7 | 1 | 8 |  |

^a^surgical site infection.

Supplementary Table 4: P-values rom Wilcoxon rank sum test to assess impact of various factors on complication rates in all TPLO-only (n=57) and TPLO+ARS (n=28) cases.

| **Complications** | **Factors** | | | | |
| --- | --- | --- | --- | --- | --- |
|  | Anesthesia  time (min)^a^ | Surgery  time (min)^a^ | Arthroscopy  (Yes vs No) | ARS placement  (Yes vs No) | Plate type (3.5mm  standard vs broad) |
| Major complication | 0.38 | 0.55 | 0.48 | 1.00 | 1.00 |
| Minor complication | 0.92 | 0.76 | 0.70 | 0.42 | 0.70 |

^a^minutes
